# Fundamentals for forage crop breeding and seed production in Russia

**DOI:** 10.18699/VJ21.044

**Published:** 2021-07

**Authors:** V.M. Kosolapov, V.I. Cherniavskih, S.I. Kostenko

**Affiliations:** Federal Williams Research Center of Forage Production and Agroecology, Lobnya, Moscow region, Russia; Federal Williams Research Center of Forage Production and Agroecology, Lobnya, Moscow region, Russia; Federal Williams Research Center of Forage Production and Agroecology, Lobnya, Moscow region, Russia

**Keywords:** fodder crops, plant genetic resources, source material for breeding, molecular certificate, DNA markers, кормовые культуры, генетические ресурсы растений, исходный материал для селекции, молекулярно-генетический паспорт, ДНК-маркеры

## Abstract

Plant breeding and seed production of new generation fodder crops is the groundwork for creating a fodder base for livestock production in sufficient quantities. The Federal Williams Research Center of Forage Production and Agroecology founded in 2018 based on of the All-Russia Williams Fodder Research Institute and other scientific institutions is the largest and most comprehensive center in the field of food production. It develops new techniques and methods for creating initial seed material based on a wide use of genetics, biotechnology, microbiology, immunology, ecology, biogeocenology, and cell selection. During the existence of the Fodder Research Institute and its experimental stations, up to 300 varieties of feed crops were created, which occupied leading positions in the production of fodder in meadows, pastures, and hayfields. Eighty-five modern varieties of fodder crops of the latest generation are widely used and zoned in all regions of Russia. However, the destroyed system of elite and commercial seed production does not allow these varieties to take their rightful place in fodder production, and the market still possesses a large share of non-varietal and mass scale reproduction seeds. In addition, imported seeds brought to the Russian market are often disguised as lawn varieties to reduce the cost and simplify their entry to the market. In this way, 107 varieties of winter ryegrass (Lolium perenne L.), 47 varieties of cane fescue (Festuca arundinacea Schreb.), 21 varieties of creeping clover (Trifolium repens L.), etc. appeared in Russia. In such circumstances, the attention of the Williams Center is focused on the development of techniques and methods for creating fundamentally new varieties based on its own research in genetics, biotechnology, immunology, and ecological selection. Much attention is paid to expanding the network of research stations throughout Russia in order to revive the system of elite seed growing, especially in the regions with the most favorable climate for growing seeds of particular crops. A seed production center was organized as a branch of the Williams Center at the end of 2020. In the future, it is planned to create a united coordinated interdepartmental complex for the breeding of fodder crops in accordance with the regional needs of animal husbandry

## Abstract (ru)

Селекция и семеноводство кормовых культур являются фундаментом создания кормовой базы для производства животноводческой продукции высокого качества и в требуемом количестве. Федеральный научный центр кормопроизводства и агроэкологии им. В.Р. Вильямса (ФНЦ «ВИК им. В.Р. Вильямса»), созданный в 2018 г. на базе Всероссийского НИИ кормов им. В.Р. Вильямса и ряда других научных учреждений, – наиболее крупный центр в области кормопроизводства – разрабатывает новые приемы и методы создания селекционного материала на основе широкого применения генетики, биотехнологии, микробиологии, иммунологии, экологии, биогеоценологии, клеточной селекции. Было выведено около 300 сортов кормовых культур, которые занимали в СССР и занимают в Российской Федерации основные площади под этими культурами на лугах, пастбищах и сенокосах. Современные 85 сортов кормовых культур нового поколения нашли широкое применение и районированы во всех регионах России. Но разрушенная в стране система элитного и товарного семеноводства не позволяет этим сортам быть на положенном им месте в производстве кормов, так как на рынке еще велика доля несортовых семян и семян массовых репродукций. К тому же началась экспансия импортных сортов, замаскированных под сорта газонных трав для удешевления и упрощения выхода их на российский рынок. Таким образом на наш рынок попали 107 сортов райграса пастбищного (Lolium perenne L.), 47 сортов овсяницы тростниковой (Festuca arundinacea Schreb.), 21 сорт клевера ползучего (Trifolium repens L.) и т. д. Основное внимание ФНЦ «ВИК им. В.Р. Вильямса» направлено на создание приемов и методов получения принципиально новых сортов на базе собственных разработок в области генетики, биотехнологии, иммунологии и экологической селекции. Расширяется сеть опорных пунктов по всей территории страны, воссоздается система элитного семеноводства с учетом использования регионов с наиболее благоприятным климатом для семеноводства отдельных культур. В конце 2020 г. на базе ФНЦ «ВИК им. В.Р. Вильямса» создан селекционно-семеноводческий центр. В дальнейшем планируется сформировать единый межведомственный комплекс по селекции кормовых культур, работающий по скоординированной программе согласно региональным потребностям животноводства.

## Introduction

Fodder crop breeding and seed industry regain their paramount significance. Animal husbandry cannot be developed without well-handled fodder supply. In addition to the main function of fodder crops, which is the production of bulk feed for animals, they play other roles, no less important and sometimes outstanding: the formation of stable ecosystemic agrarian landscapes, improvement of performance and phytosanitary state of cultivated lands, soil protection from erosion by water and wind, and aesthetic function, as they form the basis for favorable living environment in cities. 

Genetic resources of plants attract the attention of scientists throughout the world. They raise not only biological but also political challenges, being involved in the competition not only in the academic community but also among multinational food-producing corporations (Genetic Resources…, 2016). Breeding programs for fodder plants rest on the gene pool as the main source of commercially significant traits. The role of the initial material and the geographic distribution of plant genes is the basic tenet in breeding science (Vavilov, 1987). 

The diversity of forage plant species, varieties, and ecotypes, including natural populations, permits breedersto involve diverse breeding material and raise cultivars for various purposes. Much attention is paid to the involvement of forage plant resources. In annual expeditions collecting forage plants, the gene pool department of the Federal Williams Research Center of Forage Production and Agroecology (hereafter the Williams Center) has assembled a collection of more than 7,000 accessions for long-term storage. Genetic resources of wild forage plants have been collected and examined in many regions of Russia: the Kirov oblast, Udmurtia, Tatarstan, Karelia, Altai, the Ryazan oblast, lower reaches of the Don River, and along the Oka River floodplain. The expedition route lengths in recent years have totaled over 20,000 km (Trofimova et al., 2019). 

In addition to the mobilization, collection, and use of genotypes from natural vegetation, new materials for breeding are being produced by using mutagenesis, somaclonal variants, polyploidy, hybridization, and synthetic hybrid populations. Biotechnological methods for clonal micropropagation and somatic hybridization are in broad use. Cell selection is applied to breeding for fungus resistance and tolerance of some adverse environmental factors. The breeding material is tested against artificial infectious backgrounds and under laboratory conditions. Work on genetic markers is being conducted, and it brought about the Soleustoichivaya salt-tolerant alfalfa variety and a series of new-generation red clover (Trifolium pratense L.) varieties (Kosolapov et al., 2019). 

The main direction in the modern fodder breeding strategy is targeting. It implies the necessity of a system involving a diversity of varieties whose climatic and ecological differentiation would make them fit for specific conditions of each region. 

## Ecological and geographical approach to the creation of new varieties of forage crops

The combined biogeocenotical approach is implemented via phytocenotic selection, based on the doctrine of competitive and neutral interactions among plants; edaphic selection, based on the response of plants to the physicochemical and biochemical features of the edaphic environment; and symbiotic selection, based on mutually beneficial interaction of plants with nitrogen-fixing microbes (Shamsutdinov, 2010). 

By now, over 740 fodder plant varieties have been raised in Russia, of which over 240 – at the Williams Center. Eighty-five new generation varieties have become the most widespread (Kosolapov et al., 2019).

A significant part in fodder production and raise of high-performance agrophytocenoses is assigned to clover. Breeders of the Williams Center have raised over 20 red clover (T. pratense L.) varieties: Mars, VIK 7, Tetra VIK, VIK 84, Rannii 2, Trio, Dedinovskii 5, Zarya, VIK 77, Topaz, TOS 870, Orlik, Stodolich, Ratibor, Altyn, Dobrynya, Meteor, Mariya, Pamyati Lisitsina, Pamyati Burlakii, etc. They form the required set of zoned varieties for all Russian regions. A series of alsike clover (Trifolium hybridum L.) varieties have been raised (Kosolapov et al., 2019). This species is important in regions with poor heat supply and acidic waterlogged soils. The varieties Marusinskii 488, Pervenets, and Mayak may be of great importance on peaty soils of northern Russia. 

With the intense development of organic agriculture and high-quality farming products, meadow forage production with the series of varieties including Smena, VIK 70, Lugovik, etc., swards can be formed in all zones of Russia where it is practicable. 

Alfalfa (Medicago L.) is an important fodder crop forming the base of high fodder production and high-tech animal husbandry in the world (Chernyavskikh et al., 2012). The most urgent task is its expansion to the north, to the vast Nonchernozem Belt, and other regions with a short growing season (Urals and Siberia), acidic soils, and the flushing soil regime. Varieties sustaining on pastures and salinized soils are demanded. 

Fundamentally new breeding approaches and methods brought about many unique varieties: Lada, Pastbishchnaya 88, Lugovaya 67, Selena, Soleustoichivaya, Sonata, Nadezhda, Nakhodka, Galiya, and Vega 87, the last variety being the most widespread in Russia. A new cultivar of subspecies varia, Agniya, shows a high level of symbiotrophism, which allows accumulation of 270–300 kg of fixed nitrogen per hectare

Fodder grasses are the base for fodder phytocenoses on waterlogged, acidic, and cold soils as well as under the conditions of erosion by water on slopes and by wind on light soils. 

With the grass collection of the Williams Center, cultivars for all regions of Russia can be raised. Scientists of the Williams Center have bred varieties of timothy (Phleum pratense) (VIK 9, VIK 85, and VIK 911), winter ryegrass (Lolium perenne L.) (VIK 66, Duet, Tsna, and others), tall fescue (Festuca arundinacea Schreb.) (Lira), festulolium (×Festulolium F. Aschers. et Graebn.) (VIK 90, Fest, and Alegro); meadow fescue (Festuca pratensis Huds.) (VIK 5, Kvarta, Binara, Dedinovskaya 8, Krasnopoimskaya 92, and others), Kentucky bluegrass (Poa pratensis L.) (Tambovets, Pobeda, and Dar), Hungarian brome (Bromopsis inermis (Leyss.) Holub) (Morshanskii 760, Fakel’nyi, Voronezhskii 17, Pavlovskii 22/05, and others), cock’s foot (Dactylis glomerata L.) (VIK 61 and Dedinovskaya 4), crested wheatgrass (Agropyron pectinatum (M. Bieb.) P. Beauv.) (Pavlovskii 12), red fescue (Festuca rubra L.), Regel’s tall fescue (Festuca regeliana Pavlov), black bent (Agrostisgigantea Roth), etc. (State Register…, 2021). The breeding process at the Williams Center involves the creation of varietal–microbial consortia, improvement of the ability of varieties to fix atmospheric nitrogen, and improvement of the phosphorus-mobilizing potential. The Williams Center works intensively on the breeding of lupine (over 20 varieties), vetch, and cruciferous vegetables.

Accessions are being tested for resistance to major pests. Data for the database for long-term phytosanitary monitoring of major fodder crop diseases have been obtained. New fungal pathogen isolates have been isolated from the root microbiota of red clover (T. pratense L.), grasses, and vetch (Vicia L.) The breeding and growing of varieties resistant to adverse biotic and abiotic environmental factors contributes much to the control of the phytosanitary state of farming ecosystems. 

The breeding of arid crops holds a special place in the Williams Center. It is of prime importance with regard to the current climatic changes, the expansion of arid, blown, and salt-affected arable lands, and desertization. A prominent scholar school on arid crop breeding exists at the Williams Center. New varieties of Haloxylon aphyllum (Minkw.) Iljin, Kochia prostrata (L.) Schrad., Salsola orientalis S.G. Gmel., Salsola subaphylla C.A. Mey, Camphorosma lessingii Litv., Poa bulbosa L., Elymus racemosus Lam., and others are targeted at the formation of year-round pastures. The scientific substantiation of the environment-forming role of halophytic plants in vegetative reclamation, physical loosening of the soil pan and improvement of salinized soil drainage conditions by plant roots, organic matter accumulation for the nutrition of microbes in aridic and salinized plant ecosystems, salt lowering in the root layer of halophytes, etc. have been developed. 

## Main directions and ways of development breeding of forage crops in Russia 

Another important line of inquiry is the development of a DNA marker-based system for molecular tagging aimed at the genetic identification and certification of fodder crop varieties (Chesnokov et al., 2019). 

**Fig. 1.**
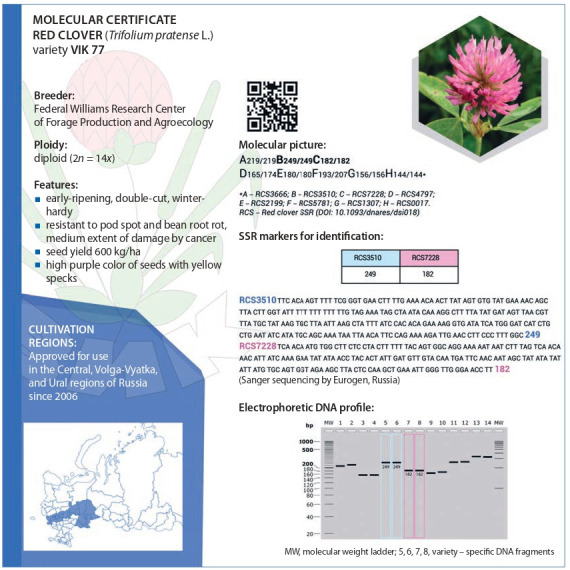
An exemplary molecular certificate: red clover (T. pratense L.) variety VIK 77.

The Laboratory of Molecular Studies of Fodder Crops was founded in the Williams Center in 2019. Its primary task is the development of molecular tagging systems for cultivar certification, DNA tagging of traits essential for breeding, and study of the genetic variability of wild and cultivated fodder plant species (Klimenko et al., 2020). The results indicate that the identification of cultivars by DNA markers improves the protection of patents as intellectual property items and shortens the time for the development of high-performance fodder crop varieties resistant to adverse environmental factors. A molecular certificate form for crops based on SSR markers has been designed (Fig. 1). Comparison of a sample with a standard DNA certificate will help in genetic identification, testing varietal purity, and testing the compliance of seed material with the variety certificate.

**Fig. 2.**
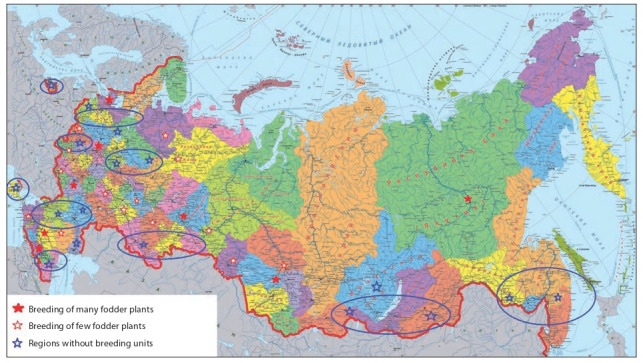
Regions most promising for the creation of field stations for fodder crop breeding.

For successful breeding of fodder crops, special attention should be paid to the activity of existing breeding units, the restoration of closed ones, and the creation of new ones in various regions of Russia. This notion is no novelty, but its effectiveness in ecological tests of new forms has been proven. All that is left is to remember our recent past. It is increasingly important with regard to the ongoing rapid global climatic changes (Chernyavskikh et al., 2012). The formation of the field station network should be continued. Fundamentals for the geographic distribution of crops have been developed (Fig. 2). 

**Fig. 3.**
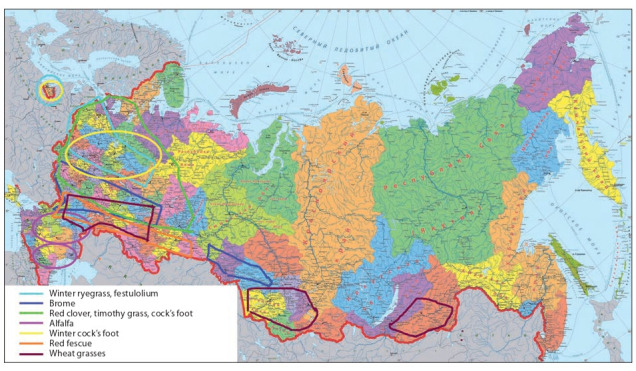
The best regions for growing various fodder crops.

The distribution of these field stations correlates with regions of the most favorable location of fodder crop seed industry and the greatest seed yields (Fig. 3). It is a complicated piece of work, but the activity of the creative association of breeders (CAB) “Clover” has been a good practice. The collaboration of its members produced 12 newgeneration winter-hardy high-performance varieties of red clover (T. pratense L.):

- Trio (Williams Center + Federal Agrarian Research Center for the Far Northeast);
- Meteor (Siberian Federal Research Center of Agrobiotechnologies + Williams Center);
- TOS 870 (Buryatian State Agricultural Academy + Williams Center);
- Ratibor (Federal Agrarian Research Center for the Far Northeast + Williams Center);
- Orlik (Federal Scientific Center of Legumes and Groat Crops + Williams Center);
- Altyn (Williams Center + Morshansk Breeding Station);
- Mariya (Buryatian State Agricultural Academy + Williams Center);
- Pamyati Lisitsyna (Federal Scientific Center of Legumes and Groat Crops + Siberian Federal Research Center of Agrobiotechnologies + Williams Center);
- SOZh (Buryatian State Agricultural Academy + Williams Center);
- VIK 84 (Williams Center + Moscow Breeding Station + Nemchinovka Federal Research Center);
- Dobrynya (Williams Center + Nemchinovka Federal Research Center);
- Prima (Siberian Federal Research Center of Agrobiotechnologies + Williams Center).

Five more red clover varieties are under official tests in Russia and Belarus (Ecological Selection…, 2012). 

## Seed production of forage crops: state of the art and prospects

Deeper insight into the current state and potential of fodder crop breeding and seed industry shows that seed farming is in the most trouble. The state of breeding in Russia is satisfactory: many varieties have been developed, and some of them are outstanding, but primary and elite seed production are rated low. Unfortunately, the cooperation between fodder crop breeding and seed industry presents a lingering problem in Russia. The remark made by N.I. Vavilov at the All-Russia Conference on Fodder Plant Breeding and Seed Industry still sounds relevant: “We should combine our research with production, not only without descending from theoretical heights but raising them even higher. We should manage the work of all our facilities, including botanical nurseries, so as to arrange a pipeline towards production, reproduction, and seed industry.” (Vavilov, 1935, p. 3). 

The major issues are poor funding, obsolete machines, aging staff, uncertainty of intellectual property, etc. With the breakup of the Soviet Union, the interactions in the breeding and seed industry of fodder crops, in particular, perennial plants, based on the clear mediation system of breeding research enterprises, elite seed farms (group one seed farms), and farms producing seeds for reproduction (group two farms) were disrupted. The fancied market system, based on the economic concern of all participants, did not come into being. No prerequisites for the development of an efficient interaction system between breeding and seed production have emerged within the elapsed 30 years.

The succession breeding–primary seed production–commercial seed production–commercial farming experiences stagnation, most pronounced in the first two links. The cause is apparent: chronic underfunding because of no rapid remuneration. It is aggravated by the economic pressure of multinational companies, which are key stakeholders in the world seed and food market. For instance, 107 varieties of winter ryegrass (L. perenne L.), 47 varieties of cane fescue (F. arundinacea Schreb.), and 21 varieties of creeping clover (T. repens L.) were enlisted into “The State Register of Selection Achievements Authorized for Use for Production Purposes” (2021) as lawn grasses to make it simpler and cheaper to push them to the market. In this way, they came to the Russian market through a hole in Russian regulations to be sold as fodder crops. In most cases, such swards survived till the nearest winter. This situation not only jeopardizes the breeding research institutions and education of students and postgraduates. The very Russia and Russian nation are menaced (Kosolapov et al., 2012). The premise “we will buy everything” does not work. We will not. Fodder scientists and breeders are aware of the menace. Producers in major agrarian enterprises are also coming to this awareness. 

**Fig. 4.**
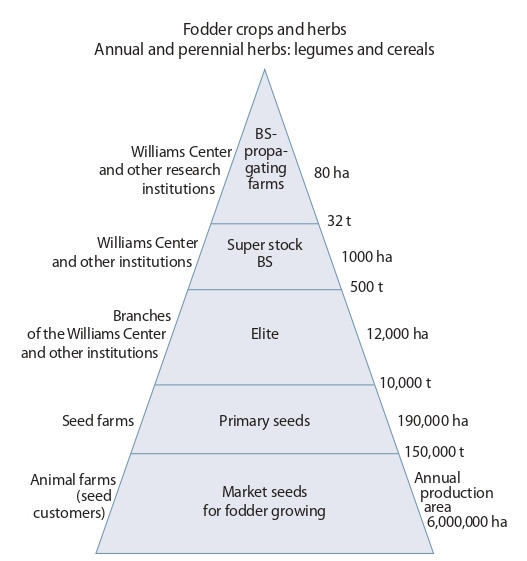
Structure of fodder crop production. BS – breeder seed.

With the groundwork in hand, we can rehabilitate the production of elite fodder crop seeds by collaboration of research institutions belonging to various departments and commercial enterprises, raise sets of appropriate varieties for most regions of Russia, and provide enough high-quality seeds to animal farming industry (Fig. 4). 

Breeding science and primary seed production demand funding. Transparent interaction among governmental institutions, breeding institutes, private seed-producing enterprises, and agricultural commodity producers is essential. 

## Conclusion

While having a potent academic base, fodder breeding and seed industry in Russia need support from federal and regional authorities, large holding groups, and large farms. This is true for breeding all crops, this is our future, which we can make present in a short while

We have a stepping stone to solving the tasks posed by fodder crop breeding and seed industry. Academic schools of experts in breeding alfalfa, clover, vetch, grasses, and arid crops exist and continue their development. They prime groups of followers, carry basic works on donor identification, and fill pre-breeding collections. A method for identification and certification of fodder crop varieties has been developed in order to test the varietal purity and conformity to varietal standards and to improve seed production efficiency. However, issues related to the system of elite seed production are still to be resolved, and they should be in the focus of science, production, and business.

## Conflict of interest

The authors declare no conflict of interest.
